# Supplementary material for: Transcriptome analysis of the growth performance of hybrid mandarin fish after food conversion
Source: PLoS One. 2020 Oct 9;15(10):e0240308. doi: 10.1371/journal.pone.0240308 (PMC7546499; doi:10.1371/journal.pone.0240308)
Supplement: S1 Table — (DOC) [file pone.0240308.s001.doc]

**S1 Table. The detailed sample information of hybrid mandarin fish.**

|  | **Number** | **TL(cm)** | **BL(cm)** | **BH(cm)** | **W(g)** |
| --- | --- | --- | --- | --- | --- |
| **F** | F1 | 22.2 | 18.7 | 6.6 | 167.37 |
|  | F2 | 20.6 | 17.5 | 6.2 | 155.79 |
|  | F3 | 21.2 | 17.8 | 6.1 | 154.30 |
|  | F4 | 21.6 | 18.3 | 6.3 | 154.08 |
|  | F5 | 20.8 | 17.6 | 6.3 | 143.81 |
|  | Mean | 21.28±0.57^b^ | 17.98±0.45^b^ | 6.3±0.17^b^ | 155.07±7.49^b^ |
| **S** | S1 | 14.3 | 12.1 | 4.5 | 49.49 |
|  | S2 | 15.3 | 12.9 | 4.1 | 58.14 |
|  | S3 | 15.6 | 13.2 | 4.4 | 58.38 |
|  | S4 | 14.5 | 12.2 | 4.2 | 49.27 |
|  | S5 | 14.7 | 12.1 | 4.5 | 53.88 |
|  | Mean | 14.88±0.49^a^ | 12.50±0.46^a^ | 4.34±0.16^a^ | 53.83±3.97^a^ |

F, Fast group; S, Slow group; TL, Total Length; BL, Body Length; BH, Body Height; BW, Body Weight. Means in same column with different superscripts were very significantly different (P < 0.05).
