# Supplementary material for: Transcriptome analysis of the growth performance of hybrid mandarin fish after food conversion
Source: PLoS One. 2020 Oct 9;15(10):e0240308. doi: 10.1371/journal.pone.0240308 (PMC7546499; doi:10.1371/journal.pone.0240308)
Supplement: S2 Table — (DOC) [file pone.0240308.s002.doc]

**S2 Table. The domestication rate and survival rate of hybrid mandarin fish.**

| **Groups** | **TN** | **30****D****SN** | **60DSN** | **90DSN** | **SR (%)** |
| --- | --- | --- | --- | --- | --- |
| **1** | 200 | 137 | 119 | 109 | 55.00 |
| **2** | 200 | 151 | 135 | 126 | 63.00 |
| **3** | 200 | 155 | 131 | 123 | 62.00 |
| **Mean** | 200 | 147.67 | 128.33 | 119.33 | 60.00±3.56 |

TL: Total number; DSN: Day Survival Number; SR: Survival Rate.
