# Supplementary material for: Transcriptome analysis of the growth performance of hybrid mandarin fish after food conversion
Source: PLoS One. 2020 Oct 9;15(10):e0240308. doi: 10.1371/journal.pone.0240308 (PMC7546499; doi:10.1371/journal.pone.0240308)
Supplement: S3 Table — (DOC) [file pone.0240308.s003.doc]

**Table S3. The data of DEGs in the S vs. F groups of hybrid mandarin fish.**

| **Pathway** | **KO_id** | **KO_name** | **KO_id** | **KO_name** | **KO_id** | **KO_name** |
| --- | --- | --- | --- | --- | --- | --- |
| **Fatty acid biosynthesis (4)** | K11262 | ACACA | K01946 | ACACB | K15013 | ACSBG |
|  | K00665 | FASN |  |  |  |  |
| **Collecting duct acid secretion (6)** | K02147 | ATP6B | K02150 | ATP6E | K02154 | ATP6N |
|  | K18245 | CA2 | K13627 | KCC4 | K06573 | SLC4A1 |
| **Cell cycle (21)** | K06629 | ASK | K02178 | BUB1 | K06637 | BUB1B |
|  | K06627 | CCNA | K05868 | CCNB | K03363 | CDC20 |
|  | K05866 | CDC25B | K06628 | CDC45 | K10151 | CCND2 |
|  | K06626 | CCNE | K02087 | CDK1 | K06618 | DMBT1 |
|  | K02365 | ESP1 | K04402 | GADD45 | K06638 | MAD1L |
|  | K02537 | MAD2 | K04377 | MYC | K06631 | PLK1 |
|  | K03875 | SKP2 | K04500 | SMAD2_3 | K08866 | TTK |
| **IGF system (1)** | --- | IGFBP1 |  |  |  |  |
