# Supplementary material for: Transcriptome analysis of the growth performance of hybrid mandarin fish after food conversion
Source: PLoS One. 2020 Oct 9;15(10):e0240308. doi: 10.1371/journal.pone.0240308 (PMC7546499; doi:10.1371/journal.pone.0240308)
Supplement: S4 Table — (DOC) [file pone.0240308.s004.doc]

**S4 Table. The SNP types information of hybrid mandarin fish.**

| **Sample** | **Homo SNP** | **Hete SNP** | **Total** |
| --- | --- | --- | --- |
| **F1** | 260838 | 194243 | 455081 |
| **F2** | 235105 | 225585 | 460690 |
| **F3** | 288644 | 179430 | 468074 |
| **F4** | 292952 | 173280 | 466232 |
| **F5** | 302807 | 158540 | 461347 |
| **F** | 1380346 | 931078 | 2311424 |
| **S1** | 293337 | 165787 | 459124 |
| **S2** | 267013 | 179727 | 446740 |
| **S3** | 235233 | 224779 | 460012 |
| **S4** | 239580 | 178174 | 417754 |
| **S5** | 277307 | 153273 | 430580 |
| **S** | 1312470 | 901740 | 2214210 |
