# Supplementary figures and images for: Transcriptome analysis of the growth performance of hybrid mandarin fish after food conversion
Source: PLoS One. 2020 Oct 9;15(10):e0240308. doi: 10.1371/journal.pone.0240308 (PMC7546499; doi:10.1371/journal.pone.0240308)

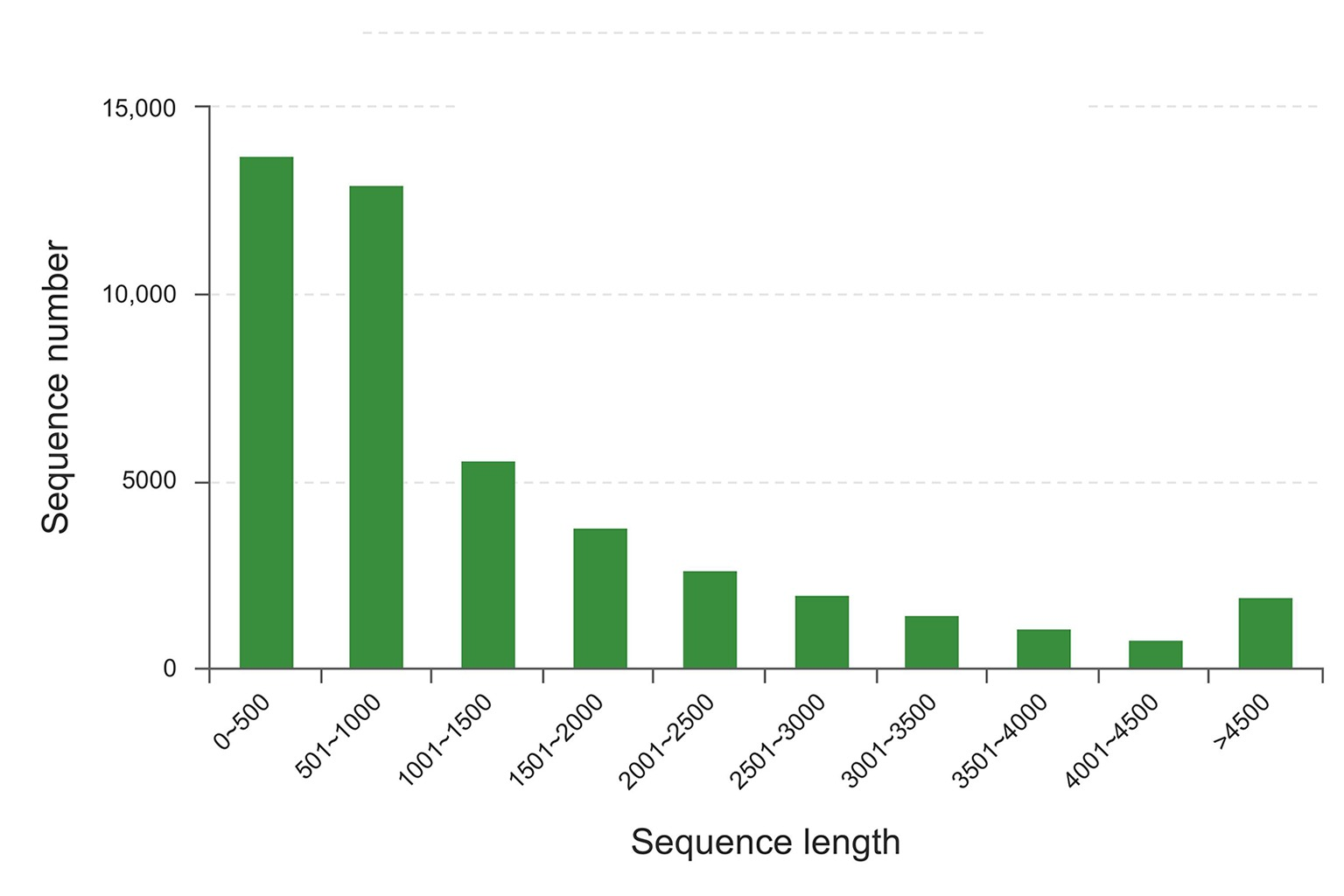

Supplement: S1 Fig — (TIF) [file pone.0240308.s006.tif]

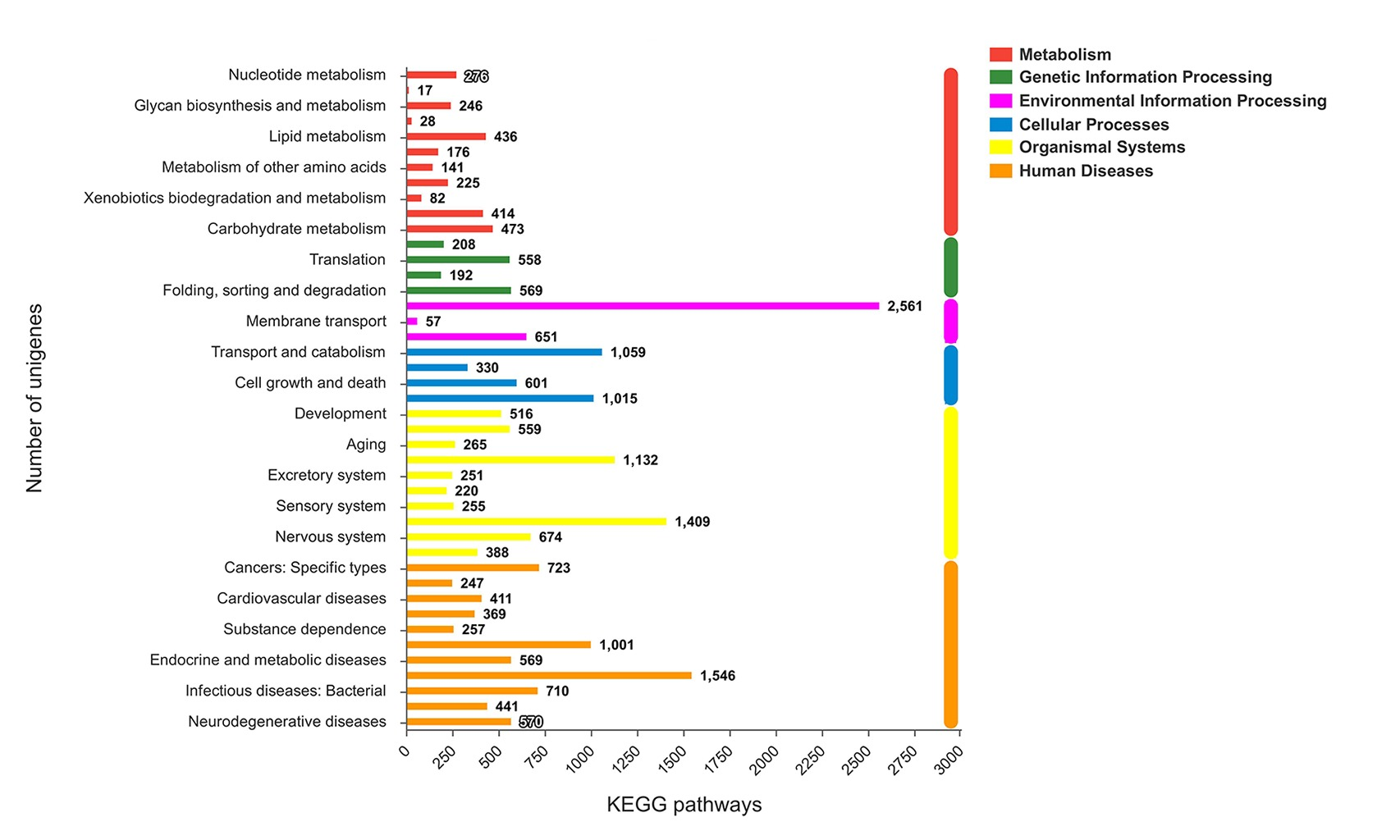

Supplement: S2 Fig — (TIF) [file pone.0240308.s007.tif]

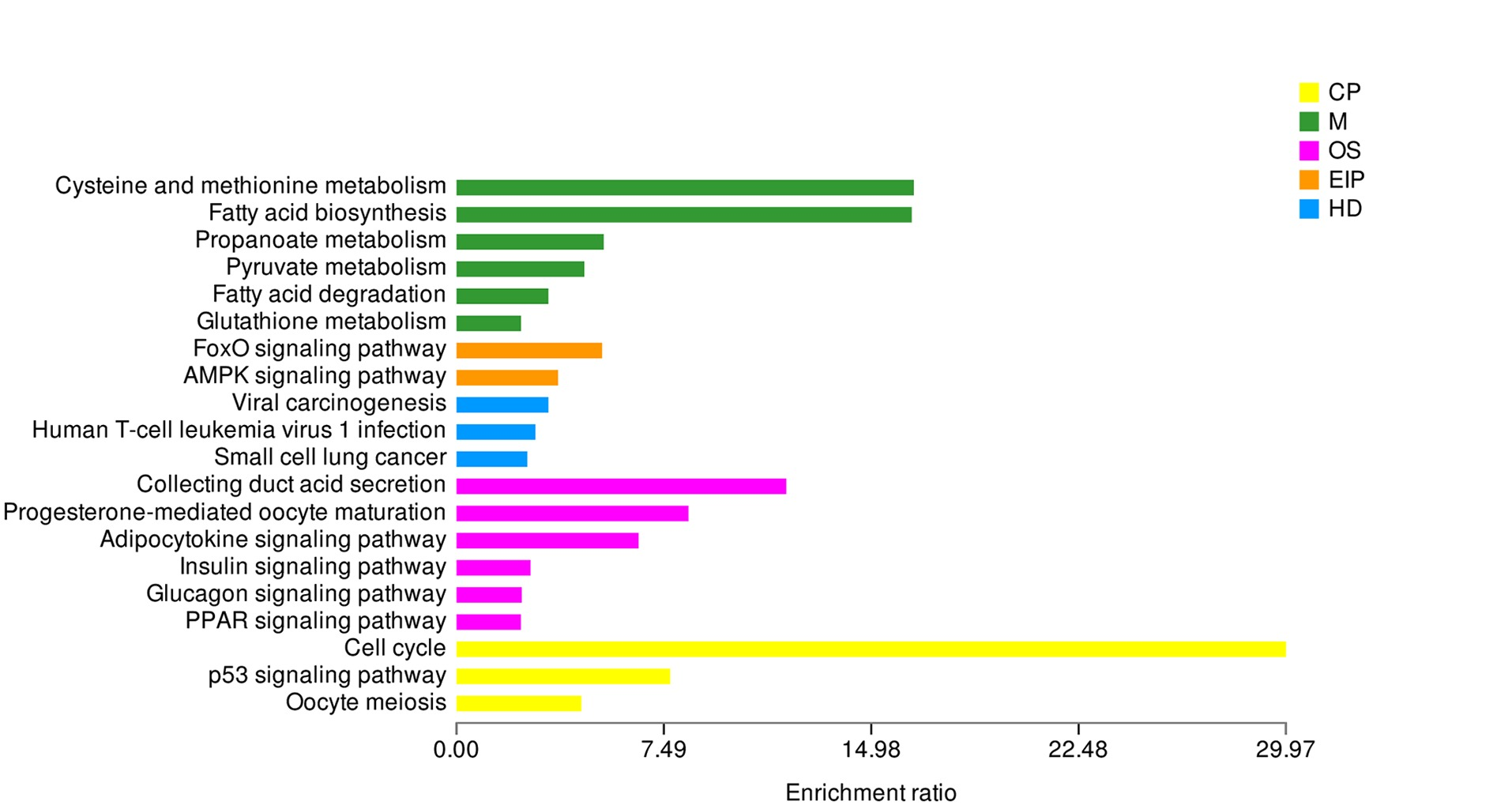

Supplement: S3 Fig — The y-axis represents the KEGG pathway, and the x-axis represents the significance level of enrichment. Different colors represent 7 branches of the KEGG metabolic pathway, containing cellular processes (CP), metabolism (M), organismal systems (OS), environmental information processing (EIP), human diseases (HD). (TIF) [file pone.0240308.s008.tif]
