# Supplementary material for: Transcriptome analysis of the growth performance of hybrid mandarin fish after food conversion
Source: PLoS One. 2020 Oct 9;15(10):e0240308. doi: 10.1371/journal.pone.0240308 (PMC7546499; doi:10.1371/journal.pone.0240308)
Supplement: Attachment — Submitted filename: Reviewer comments.docx [file pone.0240308.s009.docx]

**Reviewer comments**

The manuscript (PONE-D-20-17403) entitled "**Transcriptome analysis of the growth performance of hybrid mandarin fish after food conversion**" which submitted to PLOS ONE by Guan WZ et al. has been reviewed.

The author used artificial feed to raise hybrid mandarin fish for 3 months, and measured the food conversion rate, survival rate, total length, and body weight of the hybrid fish, absolute length and weight gain rate. Then use RNA-Seq to identify potential genes involved in the pathways activated growth performance. For example: fatty acid biosynthesis (FASN and ACACB), collection of duct acid secretion (ATP6E and KCC4), cell cycle (CDC20 and CCNB) and insulin-like growth factor (insulin) -like growth factor, IGF) system (IGFBP1) and other pathways may be related to the growth of mandarin fish. Finally, it was also found in fast-growth fish, more potential single nucleotide polymorphisms (SNPs) were detected than in slow-growing fish. However, there are still some problems with the experimental design and content of this manuscript, which need to be supplemented and further explained and clarified by the author.

Q1-

What is the breeding pattern and origin of “the fifth generation hybrid fish” mentioned in this manuscript material and method? Regarding the source and definition of breeding biological materials, it is necessary to ask the author to add more details in the manuscript.

Q2-

What is the control group for the measurement of food conversion rate, survival rate, total length, body weight, absolute length, and weight gain rate of hybrid fish? In theory, there should be non-hybrid pure breeds or hybrids of different generations for comparison. To use this to emphasize the advantages of hybrids, the author should add *Siniperca chuatsi* and *Siniperca scherzeri* two uncrossed pure breeds. It is the control group (triple group) and compares various growth and survival values.

Q3-

P156, Group1-3 in Figure 1(A) refers to the number of deaths at 0, 30, and 90 days in the triple-repeat population of the hybrid population. Why is there no 60-day data? It is impossible to understand why the author presented the triple-duplicate data of a hybrid species alone. What is the significance?

Q4-

Figure 1(C) and Figure 1(D) are the growth data of how many months old? It is suggested that the author should make up and give details.

Q5-

In order to make it easier for readers to understand, basically the author should provide the growth data or growth curve of the F and S groups at each month of age?

Q6-

Although the author mentioned in the manuscript that more potential single nucleotide polymorphisms (SNPs) are detected in fast-growth fish than in slow-growing fish. However, the author did not actually provide such comparative figures or tables in the manuscript.

Q7-

There are more potential single nucleotide polymorphisms (SNPs) found in fast-growing fish. These nucleotide variation site markers are mainly located on those important growth genes. The authors also found in this result. No relevant research diagrams or tables are provided in this manuscript.
